# How ready is the system to deliver primary healthcare? Results of a primary health facility assessment in Enugu State, Nigeria

**DOI:** 10.1093/heapol/czaa108

**Published:** 2020-11-09

**Authors:** Adanma Ekenna, Ijeoma Uchenna Itanyi, Ugochukwu Nwokoro, Lisa R Hirschhorn, Benjamin Uzochukwu

**Affiliations:** c1 Department of Community Medicine, University of Nigeria Teaching Hospital, Enugu, Nigeria; c2 Department of Community Medicine, Institute of Public Health, College of Medicine, University of Nigeria, Enugu Campus, Nigeria; c3 Ending the HIV Epidemic Scientific Working Group, Third Coast Center for AIDS Research, Feinberg School of Medicine, Northwestern University, Chicago, IL, USA; c4 Health Systems and Policy, College of Medicine, University of Nigeria, Enugu Campus, Nigeria; c5 Consultant Public Health Physician, University of Nigeria Teaching Hospital, Enugu, Nigeria

**Keywords:** Primary healthcare, health facilities, assessment, essential drugs, drug provision, health system reform, health systems

## Abstract

Primary health centres are an effective means of achieving access to primary healthcare (PHC) in low- and middle-income countries. We assessed service availability, service readiness and factors influencing service delivery at public PHC centres in Enugu State, Nigeria. We conducted a cross-sectional study of 60 randomly selected public health centres in Enugu using the World Health Organization’s Service Availability and Readiness Assessment (SARA) survey. The most senior health worker available was interviewed using the SARA questionnaire, and an observational checklist was used for the facility assessment. None of the PHC centres surveyed had all the recommended service domains, but 52 (87%) offered at least half of the recommended service domains. Newborn care and immunization (98.3%) were the most available services across facilities, while mental health was the least available service (36.7%). None of the surveyed facilities had a functional ambulance or access to a computer on the day of the assessment. The specific-service readiness score was lowest in the non-communicable disease (NCD) area (33% in the rural health centres and 29% in the urban health centres) and NCD medicines and supplies. Availability of medicine and supplies was also low in rural PHC centres for the communicable disease area (36%) and maternal health services (38%). Basic equipment was significantly more available in urban health centres (*P* = 0.02). Urban location of facilities and the presence of a medical officer were found to be associated with having at least 50% of the recommended infrastructure / basic amenities and equipment. Continuing medical education, funding and security were identified by the health workers as key enablers of service delivery. In conclusion, despite a focus on expanding primary care in Enugu State, significant gaps exist that need to be closed for PHC to make significant contributions towards achieving universal healthcare, core to achieving the health-related Sustainable Development Goal agenda.

**KEY MESSAGES**

- Availability of recommended areas of service at primary health centres of Enugu State was variable; however, significant gaps existed in readiness to provide the care.
- Gaps in readiness to provide care were least in maternal health and greatest in non-communicable diseases (NCDs).
- The equipment needed for diagnosis of NCDs including hypertension and diabetes was available, but major gaps existed in stock of medications and staff capacity for treatment.
- Both urban and rural primary health centres had low rates of availability of essential medicines. Only one-third of the rural facilities had at least half of the recommended infrastructure/basic amenities and equipment compared with three-quarters of PHC centres in urban settings.

## Introduction

With Universal Health Coverage (UHC) as part of the Sustainable Development Goals of 2015, countries set a goal to ensure that quality health services are available to everyone, everywhere without financial hardship (WHO, 2010) by the year 2030. This goal can only be achieved through effective primary healthcare (PHC; Stigler *et al.*, 2016). PHC provides comprehensive services to the population, with both curative and preventive components. Primary health centres are an effective means of achieving access to PHC in low- and middle-income countries. These centres address geographical access (Institute of Medicine [US] Committee on Monitoring Access to Personal Health Care Services, 1993; Bitton *et al.*, 2017) and deliver primary care to the population, with an emphasis on the prevention of diseases and first contact access for primary care-amenable conditions (Barkley *et al.*, 2016).

Ensuring access to quality health services is one of the main functions of a health system. Service access includes different components: availability, affordability and acceptability. Service availability refers to the physical presence of the delivery of services and encompasses health infrastructure, core health personnel and aspects of service utilization. Service readiness is a prerequisite for service quality. Readiness is defined as the availability of components required to provide services, such as basic amenities, basic equipment, standard precautions for infection prevention, diagnostic capacity and essential medicines (World Health Organization [WHO], 2015b). These five domains define general service readiness, which refers to the overall capacity of health facilities to provide general health services. Specific-service readiness refers to the ability of health facilities to offer a specific service, and the capacity to provide that service is measured through consideration of tracer items that include trained staff, guidelines, equipment, diagnostic capacity, medicines and commodities (World Health Organization [WHO], 2015b).

PHC of an effective quality can only be delivered if health facilities are well equipped and adequately staffed, and have the right infrastructure, essential medicines and commodities available. For this reason, countries define minimum health packages, which in Nigeria is called the Ward Minimum Health Care Package. The National Primary Health Care Development Agency defines this package as a priority set of health interventions which should be provided in primary health centres on a daily basis at little or no cost to clients, supported through a government financial mechanism (Federal Ministry of Health, 2012). The package includes government-defined minimum standards of human resources, equipment, drugs, infrastructure and services for the PHC centres. It covers health interventions targeting primary care needs, including child survival (Integrated Management of Childhood Illnesses and routine immunization), safe motherhood (antenatal care, facility-based delivery, postnatal care and family planning), communicable disease control (TB, HIV and malaria), health education and community mobilization, nutrition and non-communicable disease (NCD) prevention (NPHCDA, 2010).

Given the importance of the components in meeting the goals of PHC, WHO developed a survey, the Service Availability and Readiness Assessment (SARA; WHO, 2015b), to assess the status of health facilities and generate evidence to support the planning and management of a health system. SARA has been utilized in many countries, e.g. Ethiopia, Uganda and Tanzania (Ali *et al.*, 2018; Moucheraud, 2018). It aims to generate reliable and regular information on service delivery, including on availability and readiness. Accordingly, measurement of a country’s health system's preparedness to progress towards UHC should include an assessment of the comprehensiveness of health services delivered at the PHC level (Fullman *et al.*,2018).

In low- and middle-income countries, major gaps in the capacity and delivery of basic clinical care have been documented, as well as poor quality care, including abusive care, by health providers (Okonofua *et al.*, 2018; Ntoimo *et al.*, 2019). These failings have led to poor utilization of primary healthcare facilities, particularly in the public sector (Ali *et al.*, 2018). This is true in Nigeria, where PHC centres are poorly utilized, failing to meet the goals of universal primary care (Sule *et al.*, 2008; Oyekale, 2017).

In addition, there have been recorded discrepancies between the reported and observed service availability of blood pressure apparatuses and family planning guidelines in primary care settings in some African countries (Ali *et al.*, 2018). The lack of necessary items, while not guaranteeing the provision of these services, presents a barrier to service delivery in NCD and reproductive health. Where services are available, rural facilities have been found to be less ready to offer them (Moucheraud, 2018).

### The PHC situation in Nigeria

PHC is the bedrock of the national health system. In 1978, Nigeria adopted PHC as a tool for achieving ‘Health for All’ by the year 2000, following the Alma Ata declaration, and in 1986 it selected 52 pilot local government areas (LGAs) as models for PHC implementation. This reform was funded by the federal government. From 1986 to 1990, the establishment of schools of health technology, the expansion of PHC centres to all LGAs and the devolution of responsibility for PHC to LGAs occurred. The National Primary Healthcare Development Agency (NPHCDA), established in 1992, instituted the Ward Health System and launched the Ward Minimum Health Package in 2001. The National Health Act 2014 puts PHC under the authority of the LGAs, but as this is a federal law, it is not binding on the states. This scenario constitutes the bottleneck in PHC funding in Nigeria. Regardless, the state government and its partners on occasion offer free services in key areas such as maternal and child health.

Enugu State adopted a district health system (DHS) in 2004 as a strategy to ensure PHC organizational structure and delivery. The policy was designed to address identified challenges including the unclear process of funding of PHC by the federal government, weak commitment of the local government to PHC and disagreement between the state and local government in the management of PHC (Uzochukwu et al., 2014). The DHS was a decentralization reform to ensure provision of healthcare to a geographically defined population through an integrated system linking PHC centres and secondary care facilities. The policy was also structured to develop infrastructure and health worker skills in these health facilities to improve PHC and population health. The organizational structure was also included at the state (the Policy Development and Planning Directorate and the State Health Board at the State Ministry of Health), district (the district health board) and local governmental levels, with local health authorities in charge of PHC. The reform was to be funded collectively by the state and local government authority, including partners. Enugu State began implementation in 2005, with technical support from the Partnership for Transforming Health Systems programme. Our study is designed to explore the success in implementation of a decentralized health reform policy in establishing facility capacity and readiness to provide PHC in Enugu State.

Enugu State has been implementing the DHS as opposed to the federally accepted Ward Health System implemented by other states of the federation of Nigeria, so understanding the success and challenges in translating this policy into practice has the potential to inform similar work to improve PHC. The insights derived from this study will also help inform policymakers and implementers working to achieve UHC through effective PHC, a policy priority of both state and federal governments in Nigeria.

The limited studies conducted early in the reform found challenges in improving PHC service delivery in both urban and rural centres, but there is no recent data on rural settings (Chukwuani *et al.*, 2006; Okoronkwo *et al*, 2014; Chinawa, 2015). There is no documented evidence to show if the policy has improved service availability and readiness in Enugu State 12 years after its implementation, although some researchers have reported that the DHS had the potential to foster PHC service delivery (Uzochukwu et al., 2014).

Gaps between policy, evidence and practice have been described, related to a number of factors including power roles, prioritization, transparency of goals, as well as evaluation and public accountability (Jansen *et al.*, 2010). For example, Uzochukwu et al. (2014) identified a number of gaps hindering policy implementation, including inadequate state counterpart funding for the DHS and a lack of trust between the local and state government reflected in the reluctance of local authorities to contribute to the unitary DHS fund.

Performed 12 years after the implementation of the policy to reform PHC, our study uses the SARA survey to provide evidence of the impact of the DHS policy implementation on PHC centres’ service availability and readiness in Enugu State. We also examined the potential differences between urban and rural settings and assessed provider-reported facilitators or barriers to PHC service delivery. These data are important to measure policy-to-practice gaps and to inform the implementation of new policies to improve PHC and, by extension, UHC.

## Methods

### Study area

Enugu State is one of the 36 Nigerian states, located in the eastern part of Nigeria. The state has 17 LGAs, of which 12 (70%) are rural. The state has an estimated population of 3 267 837 (National Bureau of Statistics, 2006), divided into seven health districts. It has a total of 443 public health facilities, of which 250 are primary health centres, described as comprehensive health centres, each serving a catchment population of 10 000–20 000 people.

### Study design

We conducted a descriptive cross-sectional study of the service availability of PHC centres in Enugu State and their readiness to provide core PHC functions, within the context of the DHS policy implementation.

### Study sites and sample

The study sites were primary health centres selected by simple random sampling from a sampling frame of centres in each of the seven health districts (see paragraph below for details) to achieve the minimum sample size required. The most senior health worker available at each health facility was interviewed in each sampled facility, because PHC centres may differ in staff cadre availability or distribution.

### Sample size

The sample size was determined statistically using the formula for estimation of proportion with a specified precision (Kirkwood, 2003). The required sample size, *n*, is given as p [1−p]/e2, where p is the proportion of primary health centres (21.9%) that met the stipulated service coverage in an assessment of PHC services in five Nigerian states (Christian Aid, 2015) and e is the standard error (5%). Accounting for non-response and losses with an expected response of 90%, an adjustment formula was applied as well as a correction formula for study populations <10 000 (Araoye, 2004), to give a total of 58.87 health facilities. This was rounded up to 60 primary health centres.

### Study tools

The survey instruments were adapted from WHO’s SARA (WHO, 2015a,b) to reflect the Ward Minimum Health Care Package of the National Primary Health Care Development Agency. The questionnaire had structured sections for the data collection on general and specific service availability and on qualifications and continuous medical training. Services assessed included health education and promotion; nutrition; community outreach; reproductive, maternal, newborn and childcare; communicable diseases; and selected NCDs. The questionnaire had an open-ended section for health workers to note the factors that enable and constrain service delivery in the areas of funding, continuing medical education, maintenance of equipment and any other area.

An observation checklist was used to collect data on communications, ambulance/transport for emergencies, power supply, basic client amenities, infection control, processing of equipment for reuse, healthcare waste management, supervision and basic equipment. The tools were pre-tested at a PHC centre which was not included in the final sample, to ensure they captured the correct range of services according to the national guidelines and were understandable by the respondent.

### Data collection

Data were collected from July to December 2017 by the investigator (AE) and by two university undergraduates trained by the investigator in the administration of the study tools.

### Analysis

The following domains were reported: staff, training, services, infrastructure or basic amenities, equipment, essential medicines and commodities (Table 1;  Supplementary Appendix SA1 and SA2). Basic equipment was assessed in the following areas: infection control items, sterilization equipment, laboratory items and equipment in the wards, labour and consulting rooms. One point was given for each basic amenity/infrastructure, equipment and essential medicine if it was available, functional and not expired. A score of one was given to a facility with staff who had received pre- or in-service training in each of the four purposively specified (maximum score of four) interventions during the two years preceding the survey.

**Table 1:** Ward Minimum Healthcare Package recommendations for primary health centres

Variable	Number recommended per PHC scored as 1 for available items
Infrastructure/basic amenities	40 items
Service areas	Six areas with 44 components
Staff	11 cadres
Equipment	100 items
Essential medicines and commodities	102 items
Tracer items for maternal health	16 items
Tracer items for child health	22 items
Tracer items for communicable diseases	11 items
Tracer items for NCDs	19 items
Training	Four areas
Domain score	Three domains of equipment, diagnostics, medicines and commodities
Specific-service readiness score	Four service areas of maternal, child, communicable disease and NCD services

Service-specific readiness refers to the capacity of a facility to provide a service that it offers (measured through consideration of tracer items that include trained staff, equipment, diagnostic capacity, medicines and commodities).

Descriptive statistics were used for mean scores for PHC centre readiness in the SARA methodology specific-service areas (PHC staff training, maternal health services, child health services, communicable diseases and NCDs). Specific-service readiness and domain scores were calculated according to the SARA guidelines (WHO, 2015a). The specific-service readiness score was calculated as the mean availability score of tracer items across all domains in each facility calculated for the different specific-service areas in percentage. For each domain of equipment, diagnostics, medicines and commodities, we calculated a domain score as the mean availability of noted tracer items in each domain for the selected service-specific areas (maternal health, child health, communicable diseases and NCD).

We compared mean availability between urban and rural PHC centres using the *T*-test. Fischer’s exact test was used to assess the association between availability of a medical officer in a facility and that PHC centre meeting at least half of the Ward Minimum Service Package, as well as the association between PHC centre locations (urban or rural) and meeting at least half of the Ward Minimum Service Package. A binomial logistic regression model was used to identify the independent factors influencing the availability of at least half of the Minimum Service Package in PHC centres. All statistical analysis was done at 5% level of significance. All analysis was done using SPSS version 20.0.

The open-ended data were analysed using manual content analysis (Kumar, 2014).

### Ethical considerations

Ethical approval was obtained from the Ethics Committee of the authors’ institute. All participants gave written informed consent before responding to the questionnaire.

## Results

None of the 60 surveyed PHC centres had availability of all the domains within the required service areas recommended for PHC centres, although 52 (87%) of the PHC centres had at least half of all the recommended service area domains.

The most commonly available services were newborn care and immunization in 59 (98.3%) facilities, while the least commonly available services were NCD prevention in 30 (50%), dental services in 22 (36.7) and mental services in 11 (18.3%) PHC centres (Table 2). Even when available, significant gaps were identified. While the antenatal component of maternal care was commonly available in 54–59 PHC centres, the reproductive health components were least commonly available in this service area, with only 11 (18.3%) facilities that provide intra-uterine contraceptive devices. In child health services, pneumonia and helminthiasis treatment were available in 54 (90%) of the PHC centres but only 37 (61.7%) PHC centres offered adolescent counselling and support.

**Table 2:** Service-specific availability in primary health centres of Enugu State

Service areas offered in PHCs	Sampled PHCs (*N* = 60) offering services Frequency (%)
Reproductive, maternal and newborn care	
Newborn care	59 (98.3)
Folic acid supplements	57 (95)
Delivery	57 (95)
Tetanus toxoid	56 (93.3)
Iron supplements	55 (91.7)
Monitoring for hypertension in pregnancy	54 (90)
Sulphadoxine-pyrimethamine tablets	52 (86.7)
Condom distribution	46 (76.7)
Family planning counselling	44 (73.3)
Injectable contraceptives	43 (71.7)
Oral contraceptive pills	40 (66.7)
Intra-uterine contraceptive device	28 (46.7)
Child survival	
Immunisation	59 (98.3)
Zinc supplements for diarrhoea	56 (93.3)
Worm infestation treatment	55 (91.7)
Vitamin A supplements	54 (90)
Pneumonia treatment	54 (90)
Amoxicillin for pneumonia treatment	54 (90)
Child growth monitoring	53 (88.3)
ORS for diarrhoea management	52 (86.7)
Malnutrition	50 (83.3)
Iron supplements	47 (78.3)
Adolescent counselling and support	37 (61.7)
Communicable diseases	
Malaria services	58 (96.7)
HIV testing services	41 (68.3)
Sexually Transmitted Infections services	31 (51.7)
HIV treatment	15 (25)
Tuberculosis services	13 (21.7)
Health education	59 (98.3)
Nutrition counselling	58 (96.7)
NCD prevention (hypertension, diabetes, asthma, etc.)	30 (50)
Dental services	22 (36.7)
Mental services	11 (18.3)

### General service readiness

#### Staff availability

The most common cadres of healthcare staff available at surveyed PHC centres were community health officers (95.0%), junior community health extension workers (70.0%), health attendants (60.0%), medical officers, pharmacy and laboratory technicians (46.7%) and nurses/midwifes (40.0%). Other cadres included environmental health officers (16.7%), security personnel (25.0%), medical records and general maintenance staff (26.7%). In rural PHC centres, CHEWS (87%) and attendants (60%) were the most available staff cadre, while CHEWs (100%) and nurses/midwives (93%) were most available in urban PHC centres. We found that all staff cadre were more available in urban than rural settings.

#### Staff training

Two-thirds of PHC centres had staff that had received pre-service or recent (within the last two years) in-service training in at least one of the following areas: immunization, HIV services, family planning, malaria services (Table 3). The lowest rates of training were seen in malaria services in rural PHC centres.

**Table 3:** Specific-service readiness scores at PHCs in Enugu State, Nigeria

Specific-service area	Domain	Number of tracer items available in PHCs per domain	Sum of the mean availability of each tracer item per domain in rural PHCs^a^	Specific- service readiness score^b^ (%)	Sum of the mean availability of each tracer item per domain in urban PHCs^a^	Specific- service readiness score^b^ (%)	*P*-value^c^
Training				66		73	0.46
	Immunisation	1	0.84		0.80		
	HIV diagnosis	1	0.62		0.67		
	Family planning	1	0.60		0.73		
	Malaria services	1	0.58		0.73		
Sum of values		4	2.64		2.93		
Maternal health				53		59	0.40
	Equipment	2	1.26		1.07		
	Diagnostics	7	4.51		4.26		
	Medicines and commodities	7	2.66		4.07		
Sum of values		16	8.43		9.4		
Child health				55		67	0.49
	Equipment	7	4.95		4.92		
	Diagnostics	5	1.42		2.8		
	Vaccines	3	2.39		1.74		
	Medicines and commodities	7	3.31		5.27		
Sum of values		22	12.07		14.73		
Communicable diseases				41		53	0.43
	Equipment	5	1.78		2.2		
	Diagnostics	2	0.87		1.34		
	Medicines and commodities	4	1.83		2.27		
Sum of values		11	4.48		5.81		
NCDs				33		29	0.36
	Equipment	4	3.48		3.14		
	Diagnostics	2	1.24		1.34		
	Medicines	13	1.52		0.91		
Sum of values		19	6.24		5.48		

^a^ Mean availability of tracer items in each facility = number of PHCs with tracer items in each locality / total number of PHCs in that locality (rural 45, urban 15).

^b^ Specific-service readiness score = sum of mean availability of tracer items in each facility / total number of tracer items per facility × 100.

^c^ *T*-test.

#### Infrastructure and basic amenities

Significant gaps in infrastructure were found. None of the 60 PHC centres surveyed had a functional ambulance or access to a computer with internet on the day of the assessment. Power supply through the national grid, a generator or solar supply was available in 52 (87.0%) PHC centres; however, only 11 (18.3%) had a functional facility cell-phone while 21 (35.0%) had a functional refrigerator. The rural PHC centres had more functionality when compared with the urban PHC centres in the area of safe water supply (13 [28.9] vs 21 [46.7%]) and access to flushable toilets (8 [53.3%] vs 9 [60.0%]) on the day of the assessment.

#### Basic equipment availability

No PHC centre had all the required equipment available and functional on the day of the assessment. However, there was an average of 46 out of 100 equipment items. PHC centres located in urban areas had an average of 53.5 items while the rural PHC centres had an average of 43.0 items (*P* = 0.02). More urban PHC centres (10; 66.6%) had at least 50 basic equipment available than rural PHC centres (15; 33.3%).

#### Essential medicines

No PHC centre had all the required medicines available and valid on the day of the assessment. Overall, there was an average availability of 27 out of 102 essential medicines and commodities in the surveyed facilities. The PHC centres in the urban areas had an average of 36 essential medicines and commodities in stock on the day of assessment compared with only 24 in rural PHC centres (*P* = 0.004).

#### Availability of diagnostics

On the day of the survey, 23 (51.0%) rural PHC centres had at least 50% of the required diagnostics for PHC service delivery but only five (11.0%) had all diagnostics on-site. In urban areas, only five (33.3%) of the PHC centres had all recommended diagnostics, while four (26.7%) had at least 50% of the required diagnostics.

### Specific-service readiness

In the assessment of readiness to provide services, we found that the staff in urban facilities had more opportunities for training than those in rural facilities, though the difference was not statistically significant (Table 3). The specific-service readiness score was the lowest in the NCD area (33% in the rural centres and 29% in the urban centres). Even though urban PHC centres scored higher than rural PHC centres in the other service areas, the differences were not statistically significant.

Training on immunisation services was found to be the most common in the training domain (Table 4). Our results also showed the lowest domain scores in the medicines and commodities domain for NCDs, with domain scores of 12% for rural and 11% for urban PHC centres (Table 4), and in rural centres for the communicable disease area (36%) and maternal health services (38%). We noted the availability of equipment to deliver NCD prevention despite the low availability of essential medicines required as well as the poor score (44%) in the equipment domain of the communicable diseases service area.

**Table 4:** Domain scores for specific-service areas in PHCs of Enugu State

Specific-service area	Domains	Number of tracer items available in PHCs per domain	Sum of the mean availability of each tracer item per domain in rural PHCs (%)^a^	Domain scores of specific- service in rural PHCs (%)^b^	Sum of the mean availability of each tracer item per domain in urban PHCs (%)^a^	Domain scores of specific service in urban PHCs (%)^b^
Training						
	Immunisation	1	84	84	80	80
	HIV diagnosis	1	62	62	67	67
	Family planning	1	60	60	73	73
	Malaria services	1	58	58	73	73
Maternal health						
	Equipment	2	126	63	107	70
	Diagnostics	7	451	64	426	54
	Medicines and commodities	7	266	38	407	58
Child health						
	Equipment	7	495	71	492	70
	Diagnostics	5	142	47	28	58
	Vaccines	3	239	48	174	56
	Medicines and commodities	7	331	47	527	70
Communicable diseases						
	Equipment	5	178	44	22	67
	Diagnostics	2	087	46	134	57
	Medicines and commodities	4	183	36	227	44
NCDs						
	Equipment	4	348	87	314	79
	Diagnostics	2	124	62	134	67
	Medicines	13	152	12	91	11

^a^ Mean availability of each tracer items in each domain = total number of PHCs with tracer items / total number of PHCs offering the service × 100.

^b^ Domain score = sum of mean availability of tracer items in each domain / total number of tracer items per domain × 100.

#### Urban/rural location and staffing of PHC centres

In a bivariate analysis, we examined the association between PHC centres located in urban or rural areas and the presence or absence of a medical officer and the availability of at least half of the required service areas offered in a PHC, infrastructure/basic amenities and equipment, as well as essential medicines (Table 5).

**Table 5:** Relationship between availability of a medical officer, PHC location and meeting at least half of the Minimum Service Package for PHCs

Variables	Availability of at least half of required PHC service (*N* = 44)		Availability of at least half of required essential medicines (*N* = 100)		Availability of at least half of required equipment and infrastructure/basic amenities (*N* = 140)	
	*N* (%)		*N* (%)		*N* (%)	
PHC location		*P*-value^a^		*P*-value^a^		*P*-value^a^
Rural	39 (86.7)		5 (11.1)		15 (33.3)	
Urban	14 (93.3)	0.67	5 (33.3)	0.102	11 (73.3)	0.014
Medical officer availability						
No	26 (86.7)		3 (10.0)		7 (23.3)	
Yes	27 (90.0)	1.0	7 (23.3)	0.299	19 (63.3)	0.004

^a^ Fisher’s exact test.

We found a significant relationship between the availability of at least half of the recommended infrastructure/basic amenities and equipment and the location of PHC centres in the urban areas of the state (*P* = 0.014) and the presence of a medical officer (*P* = 0.004). No significant association was identified between the availability of services recommended to be offered at PHC centres and essential medicines availability (Table 5). We also found, using a bi-nominal logistic regression model, that rural PHC centres were five times less likely to have at least half of the recommended infrastructure/basic amenities and equipment compared with urban PHC centres (AOR 0.2 [95% CI 0.05–0.7]) (Table 6).

**Table 6:** Results of a binomial logistic regression showing association between PHC location, availability of high cadre staff and availability of infrastructure and equipment

Variables	*B*	Odds ratio	95% confidence interval Lowerupper
Rural PHC location	1.705	0.182	0.049–0.668
Medical officer non-availability	1.736	0.176	0.057–0.543

### Enablers and constraints to service delivery

Respondents reported enablers of and constraints to service delivery in some major areas.

#### Continuing medical education

A major enabler noted was sponsorship for training and workshops by the Local Government, state and non-governmental organizations. A health worker noted that ‘attendance increases our zeal and knowledge’ (Health Worker 003)*.* On the other hand, other health workers noted barriers to this enabling factor to be infrequent training, late or no adverts for workshops and the omission of junior staff.

#### Ensuring essential medications

We found that 28 (47%) PHC centres sourced for medicines and commodities in the open market or through local vendors, while the drug revolving fund (DRF) scheme was run in 32 (53%) of surveyed PHC centres who received their supply from the state central shop. The DRF scheme is a system whereby monies generated from the drugs sold at PHC centres are used to replace stock to ensure the availability and efficiency of the drug management cycle. It was noted as an enabler where functional. Challenges reported included the insufficiency of the fund for running the facility. Some health workers commended the LGAs for swift and consistent supply of vaccines and other drugs.

On the other hand, constraints were noted, such as the expiration of commodities due to non-use; bad access roads and long distances to the shops for some rural PHC centres, which deterred health workers from travelling to the local or state shop to replenish stock; and the high cost of drugs at the central shop.

Stock shortages were identified as a reason for patients not utilizing the PHC centres: ‘We lack medications, which reduces the number of patients attending the PHC and also makes our work ineffective’ (Health Worker 039). In a rural PHC: ‘We have not stocked any drugs for the past four years. Therefore, we refer cases to oral/verbal therapy or counselling’ (Health Worker 009).

### Funding

A range of funding sources was reported and includes DRFs, the state ministry of health, local government, partners like UNICEF, the WHO and even some villagers. The sufficiency and consistency of these funds to sustain service delivery at PHC centres were lacking:

*No PHC will function well without adequate funding. We need this for proper maintenance of our generator, for instruments and even to pay volunteers that work with us* (Health Worker 012).

### Maintenance of equipment

Some PHC centres reported that funding for maintenance comes from non-profit organizations, state and local governments and even from the PHC. Renovations and supply of laboratory equipment have been sponsored by such partners.

Some workers responded that the constraints were the possession of old-fashioned equipment, absence of donors or partners, lack of power supply to store sterilized equipment, no staff for maintenance and poor response from the LGA:

*Some of the spoilt equipment has not been put in order despite repeated reports to the government, e.g. the solar refrigerator* (Health Worker 057).

### Staff

One major enabler in staffing was in the provision by partners of e.g. laboratory technicians, volunteer health staff and other *ad hoc* staff from the federal government’s employment initiatives, such as the N-power scheme which is an employment scheme for university graduates in select sectors of the economy. However, one barrier in staffing was noted to be the scarcity of some cadres like medical officers, health educators, registered nurses and midwives and security staff:

*We lack staff. We cannot go on leave nor can we work shifts. Even when we retire for the day, we are still called on demand. We cannot give our best because we are mostly tired. A CHEW does the work of all the required medical staff* (Health Worker 020).

### Infrastructure/basic amenities

The National Primary Health Care Development Agency provided solar-powered refrigerators to PHC centres to improve vaccination supply. Local government chairs and philanthropists also occasionally sponsor the provision of basic amenities. Where this support was lacking, we found some PHC centres which had no stable water supply, maintained high-cost commercial tankers, had broken fences and leaking roofs, lacked toilets and had waterlogged premises.

### PHC utilization

A health worker noted that villagers do not patronise the PHC centre but prefer to use the services of a traditional healer: ‘The community goes to one man for antenatal care not looking into our facility’ (Health Worker 017). However, several enablers were noted, including when there was no utilization fee (free healthcare): ‘Clients respond better whenever there are free services available’ (Health Worker 025). Staff noted the provision of free services as an enabler.

Security was also a barrier to PHC centre utilization. Most respondents noted repeated burglaries which resulted in low patient utilization in a rural PHC centre, and a lack of security staff which discourages a 24-h service. However, one health worker responded that the village health committee provides night patrol guards for the PHC centre, which has improved security and utilization.

## Discussion

This study found that all sampled PHC centres in Enugu State offered the different service areas at varying degrees but none offered all the components of each service area on the day of the assessment. These gaps between the current state of PHC services in Enugu State and the recommended standards persisted despite the development of the DHS policy to reform PHC. This observation is consistent with policy-to-practice gaps (Uzochukwu et al., 2014) in the DHS, which was meant to facilitate infrastructure development and the capacity building of health workers.

Overall, child health services were the most commonly available of all service areas both in the urban and rural PHC centres, with an emphasis on immunisation. This finding differs from other local studies which showed that treatment of ailments (100%) was the most commonly available service in Lagos State (Mohammed et al., 2010), and that malaria services (91.8%) were the most commonly available service in Anambra, Benue, Kaduna, Plateau States and the Federal Capital Territory (FCT) (Christian Aid, 2015).

We found that although PHC centres are recommended to provide mental health services, only 18% of surveyed facilities offered these services, in line with local studies (Obembe *et al.*, 2017; Anyebe et al., 2019). Mental health services at the primary care level in Nigeria consist of mental health education, advice and counselling on prevention of drugs and substance abuse, as well as early identification of mental health disorders. The low availability may suggest that the health workers are not skilled in this service provision and that knowledge about the biomedical solution to mental disorders in the communities is low (Ikwuka *et al.*, 2016). A lack of expertise may also explain the low availability of treatment of dental conditions, seen in only 37% of sampled PHC centres, as well as the poor utilization of the facilities for dental conditions by community members who would rather seek traditional care (Oke *et al.*, 2011). Community health education and awareness may be the solution to correcting these misconceptions of mental and dental health. Continuing medical education for staff can also be adopted to strengthen these areas.

Similar to the findings of a study in southwest Nigeria (Mohammed et al., 2010), we found gaps in the availability of functional ambulances, which were consistent with gaps in the referral system. The combination of a lack of both functional ambulances and dedicated cell phones means that these PHC centres have neither the means to transport severely ill patients to a relevant facility nor to call for help. This is a serious gap with grave implications and may result in poor facility utilization. Timely data management may be hindered by the absence of computers with internet access at the PHC level (Gentil et al., 2017).

Vaccines were only available in half of the facilities, whether rural or urban centres. This may be explained by the fact that most PHC centres without functional refrigerators obtain their vaccines solely on immunisation days from the LGA, implying that not having vaccines stocked in most PHC centres is to comply with the rigorous cold-chain requirement. These gaps can result in missed immunisation opportunities for children visiting the PHC centre for other reasons outside the immunisation days who may not be able to return for required vaccines. The availability of solar-powered refrigerators in PHC centres for the storage of vaccines is a potential solution that has been implemented by the NPHCDA in some centres.

In the communicable disease components, our study showed that while HIV testing services were available in 68% of PHC centres sampled, only 25% offered HIV treatment. This may indicate that community members prefer secondary or tertiary institutions for care (Onwujekwe *et al.*, 2016) and that seeking care in their communities is not appealing due to stigma. This may adversely affect the realization of the 90–90–90 UNAIDS targets in the country (Levi *et al.*, 2016). The Nigeria health system offers TB services in selected facilities (Erah and Ojieabu, 2010) and this may also explain the low availability in 22% of PHC centres studied, impeding the progress towards early case detection (Ukwaja *et al.*, 2013) and the impact of TB on HIV control (Chang *et al.*, 2015).

We saw that only 30% of PHC centres offered NCD prevention services. This low availability of NCD services is detrimental to the achievement of the Sustainable Development Goals (Kathirvel and Thakur Rapporteurs, 2018), and may be exacerbated by health workers’ lack of expertise. There was an abysmally low availability of medicines for NCDs, worse in urban areas that have more access to secondary and tertiary facilities for such clinical concerns. This finding corresponds with many others from low- and middle-income countries (Getachew *et al.*, 2017; Moucheraud, 2018). This suggests non-preparedness to tackle the rising burden of NCDs in these countries (Moucheraud, 2018).

We found that the specific-service readiness score in urban PHC centres was higher than in rural PHC centres. This contrasts with a study in 17 middle- and low-income countries in 2018 (Kanyangarara *et al.*, 2018) which showed that service availability and readiness were higher in rural facilities (29%) than in urban facilities (19%) offering emergency obstetric care services. We observed an urban–rural disparity in maternal health services of 59% and 53%, though the difference was not statistically significant. The implication may be that rural dwellers utilize PHC centres much less than urban dwellers for maternal healthcare.

Several studies agree with our findings on the constraints and enablers of service delivery (Abosede and Sholeye, 2014; Alenoghena *et al.*, 2014; Chinawa and Chinawa, 2014; Chinawa, 2015). An important factor is security. Insecurity for health workers is worse when the PHC centre is perceived as alien to the community, signifying lack of community participation.

Our study had some limitations. We could not report the service availability index for the state because only a sample of the health facilities was studied against a total facility survey of SARA; hence, results are best presented as proportions of availability. Our results on service delivery constraints and enablers were reported only by one health worker per facility and the SARA tool does not have details of their demographics to adjust for responses. We were also limited to a few areas of training for health workers in the SARA methodology. Our study may have been limited by the fact that only data on PHC centre location and availability of medical officers were fitted into our regression model because data on other potential confounders were not collected, and potential urban–rural differences were not captured. The interpretation of the results will also be tempered by the fact that the assessment was for only a day in each facility surveyed. Regardless of these minor limitations, our study is the only one known to us that has employed the SARA methodology in the assessment of PHC centres in Enugu State, Nigeria.

Universal access to quality PHC in the primary health centres in Nigeria is a critical pathway for achieving health for all; however, this access is hindered by poor implementation of policies designed to achieve UHC. This policy-to-practice gap was reflected in the lack of basic amenities in these facilities, as well as poor readiness to deliver NCD services and inadequate access to essential medicines and commodities. Continuing medical education, funding and security are key enablers of service delivery. Policies and committed supervision by the National Primary Health Care Development Agency can be targeted to close these gaps, combined with a commitment to implement policies to support this work. In 2018, Enugu State adopted the Ward Health System which prescribes a State Primary Health Care Board to oversee all PHC services and to act as a gateway to disburse funding from the National Health Insurance Scheme and the recent Basic Health Care Provision Fund. Work to monitor and support effective implementation is critical to continue the work in Enugu and more broadly to achieve quality healthcare for all.

## Supplementary data

Supplementary data are available at *Health Policy and Planning* online.

## Supplementary Material

czaa108_Supplementray_Data
