# Supplementary material for: How ready is the system to deliver primary healthcare? Results of a primary health facility assessment in Enugu State, Nigeria
Source: Health Policy Plan. 2020 Nov 9;35(Suppl 1):i97–i106. doi: 10.1093/heapol/czaa108 (PMC7649669; doi:10.1093/heapol/czaa108)
Supplement: czaa108_Supplementray_Data [file czaa108_supplementray_data.zip › czaa108-suppl_data/Appendix 1- Questionnaire.docx]

**APPENDIX 1 QUESTIONNAIRE**

Quality health is a fundamental human right. The primary health centres are widely distributed across Nigeria as a means of delivering all types of care, thereby achieving universal health coverage (UHC). While this is desired, the reality may fall short. This study seeks to find out the existing structure of primary health care and the readiness of the primary health centres in delivering UHC.

Date (dd/mm/yy): ___________________________________________________________

Name of Facility and Catchment population:____________________________________________________________

Research Identification number: ________________________________________________

How many out-patients were seen in this facility during the previous month? _____________

This questionnaire is divided into three (3) sections.

Section 1 contains questions on staffing of primary health centres.

Section 2 contains questions on services available at primary health centres.

Section 3 contains questions on constraints and challenges faced in service delivery in primary health centres.

Please tick correctly the answer that applies to you.

Section 1: STAFFING

Please indicate how many staff with each of the following qualifications are currently assigned to, employed by, or seconded to this facility.

| **No** | **Cadre** | 1. **Part time** | 1. **Full Time** | 1. **How Many Present Today** |
| --- | --- | --- | --- | --- |
| 1 | Medical Officer (if available) |  |  |  |
| 2 | Community Health Officer |  |  |  |
| 3 | Nurse/Midwife |  |  |  |
| 4 | Community Health Extension Worker, CHEW |  |  |  |
| 5 | Pharmacy technician |  |  |  |
| 6 | Junior CHEW |  |  |  |
| 7 | Environmental Officer |  |  |  |
| 8 | Medical records officer |  |  |  |
| 9 | Laboratory technician |  |  |  |
| 10 | Health attendant/Assistant |  |  |  |
| 11 | Security personnel |  |  |  |
| 12 | General maintenance staff |  |  |  |

**b. TRAINED STAFF**

Please indicate the number of staff in this facility that has received training in a number of specific interventions. Training includes pre- or in-service training **during the last two (2) years.**

| **No.** | **Question** | **1) None** | **2) Don’t know** | 1. **Number of staff** |
| --- | --- | --- | --- | --- |
| 13 | Immunization |  |  |  |
| 14 | Delivery care |  |  |  |
| 15 | Adolescent sexual and reproductive health |  |  |  |
| 16 | HIV/AIDS opportunistic infection treatment and care |  |  |  |
| 17 | HIV/AIDS counselling **only** |  |  |  |
| 18 | HIV/AIDS counselling **and** testing |  |  |  |
| 19 | HIV/AIDS antibody testing including using HIV rapid testing |  |  |  |
| 20 | Prevention of mother to child transmission (PMTCT) of HIV |  |  |  |
| 21 | Family planning |  |  |  |
| 22 | STI diagnosis and treatment |  |  |  |
| 23 | Infection control/universal precautions for handling blood and other bodily fluids |  |  |  |
| 24 | Diagnosis and treatment of malaria |  |  |  |
| 25 | Drug and supplies management |  |  |  |
| 26 | Health management information system training |  |  |  |
| 27 | Health services management |  |  |  |

**Section 2: Services available.**

1. Reproductive, maternal, newborn and child care

| No. | Type of service | 1)Yes | 2)No | 3)How many patients seen in the last month? |
| --- | --- | --- | --- | --- |
| 28 | FAMILY PLANNING  Counselling for family planning |  |  |  |
| 29 | Dispensing of male and female condoms |  |  |  |
| 30 | Dispensing of contraceptives |  |  |  |
| 31 | Dispensing injectables |  |  |  |
| 32 | Insertion of I.U.C.D. |  |  |  |
| 33 | ANTENATAL CARE  Iron Supplementation |  |  |  |
| 34 | Folic acid supplementation |  |  |  |
| 35 | Intermittent preventive treatment in pregnancy (IPTp) for malaria |  |  |  |
| 36 | Tetanus toxoid immunization |  |  |  |
| 37 | Monitoring for hypertensive disorder or pregnancy |  |  |  |
| 38 | DELIVERY  Administration of Oxytocin injection immediately after birth to all women for the prevention of post-partum haemorrhage |  |  |  |
| 39 | Monitoring and management of labour using partograph |  |  |  |
| 40 | Immediate and exclusive breastfeeding |  |  |  |
| 41 | Hygienic cord care |  |  |  |
| 42 | Thermal protection ( drying baby immediately after birth and wrapping) |  |  |  |
| 43 | Parenteral administration of antibiotics (IV or IM) for mothers |  |  |  |
| 44 | Parenteral administration of oxytocic for treatment of PPH (IVor IM) |  |  |  |
| 45 | Parenteral administration of magnesium sulphate for the management of preeclampsia and eclampsia (IV or IM) |  |  |  |
| 46 | Assisted vaginal delivery |  |  |  |
| 47 | Manual removal of placenta |  |  |  |
| 48 | Removal of retained products of conception |  |  |  |
| 49 | Neonatal resuscitation |  |  |  |
| 50 | NEW BORN CARE  Male circumcision |  |  |  |
| 51 | Support for complementary feeding |  |  |  |
| 52 | Support for weaning |  |  |  |
| 53 | CHILD CARE  Diagnose and treat child malnutrition |  |  |  |
| 54 | Provide vitamin A supplementation |  |  |  |
| 55 | Provide iron supplementation |  |  |  |
| 56 | Provide ORS to children with diarrhoea |  |  |  |
| 57 | Provide zinc supplementation to children with diarrhoea |  |  |  |
| 58 | Child growth monitoring |  |  |  |
| 59 | Treatment of pneumonia |  |  |  |
| 60 | Administration of amoxicillin for the treatment of pneumonia in children |  |  |  |
| 61 | Treatment of worm infestation |  |  |  |
|  | ADOLESCENT HEALTH |  |  |  |
| 62 | Counselling And Support |  |  |  |
| 63 | Treatment of ailments |  |  |  |

| **No.** | **IMMUNIZATION** | **1)Yes** | **2)No** | **3)How many children seen in the last month?** |
| --- | --- | --- | --- | --- |
| 64 | Routine immunization |  |  |  |
| 65 | Participation in immunization campaigns |  |  |  |
| 66 | Immunization trend follow up |  |  |  |
| 67 | Management of adverse effect following immunization |  |  |  |
| 68 | Assist in identification of acute flaccid paralysis |  |  |  |
|  |  |  |  |  |

Control of communicable diseases

| **No.** | **Type of service** | **1)Yes** | **2)No** | **3)How many patients seen in the last month?** |
| --- | --- | --- | --- | --- |
| 69 | HIV  Voluntary counselling and testing |  |  |  |
| 70 | Prescribe ART |  |  |  |
| 71 | Follow-up care for PLWA |  |  |  |
| 72 | Treatment of opportunistic infections |  |  |  |
| 73 | Community/home based care and support |  |  |  |
| 74 | TUBERCULOSIS  Preliminary diagnosis |  |  |  |
| 75 | Case tracing |  |  |  |
| 76 | Case management(for TB centres) |  |  |  |
| 77 | MALARIA  ITNs |  |  |  |
| 78 | IPT for pregnant women |  |  |  |
| 79 | Diagnosis of malaria |  |  |  |
| 80 | Treatment for children |  |  |  |
| 81 | Diagnosis of STIs |  |  |  |
| 82 | Syndromic management of STIs |  |  |  |

**Health education and community mobilization**

| **No.** | **Type of service** | 1. **Yes** | 1. **No** |
| --- | --- | --- | --- |
| 82 | Education on prevailing health issues, problems and prevention |  |  |
| 83 | Adaptation and distribution of IEC/BCC materials |  |  |
| 84 | Community mobilisation for health |  |  |
| 85 | Routine home visits |  |  |
| 86 | Community outreach |  |  |
| 87 | Promotion of personal and community hygiene |  |  |
| 88 | Advising and training community on portable water and protection of water source |  |  |
| 89 | Advice and training on safe excreta disposal |  |  |
| 90 | Advice and training on safe refuse disposal |  |  |

**Nutrition**

| **No** | **Type of service** | 1. **Yes** | 1. **No** |
| --- | --- | --- | --- |
| 91 | Identification of locally available foodstuff |  |  |
| 92 | Home, school and communal gardening |  |  |
| 93 | Nutritional education, including food hygiene |  |  |
| 94 | Screening for nutrition related problems (PEM,Anemia, Goitre) |  |  |
| 95 | Nutrition assessment e.g. MUACand identification of malnutrition in children and adults |  |  |
| 96 | Food demonstration |  |  |

**Control of Non-communicable diseases**

| No. | Types of service | 1. Yes | 1. No | 1. How many patients seen in the last month? |
| --- | --- | --- | --- | --- |
| 97 | Diagnosis of asthma, diabetes, hypertension etc |  |  |  |
| 98 | Treatment of asthma, diabetes and hypertension etc |  |  |  |
| 99 | Mental health education |  |  |  |
| 100 | Advice and counselling on prevention of drugs and substance abuse |  |  |  |
| 101 | Early identification of mental health disorder |  |  |  |
| 102 | Treatment of mild oral/dental conditions |  |  |  |

**Maintenance of PHC records**

| **No.** | **Types of service** | 1. **Yes** | 1. **No** |
| --- | --- | --- | --- |
| 103 | House numbering , community census and at risk registration |  |  |
| 104 | Issuance of adult and child health cards |  |  |
| 105 | Completion of routine and notifiable disease forms, HMIS register and summary forms |  |  |
| 106 | Basic data analysis |  |  |
| 107 | Collection of community based statistics on demography and health events including births and deaths |  |  |
| 108 | REFERRALS  Counselling and motivation for referral |  |  |
| 109 | Effecting referrals for all cases above the level and following up (2way referral) |  |  |
| 110 | Mobilising support as required from the community (VDC/WDC) to effect referrals |  |  |

**DIAGNOSTICS**

| **No.** | **Type of service** | 1. **Yes, on site** | 1. **Yes, off site** | 1. **Not available** |
| --- | --- | --- | --- | --- |
| 111 | Malaria rapid test kit |  |  |  |
| 112 | HIV rapid test kit |  |  |  |
| 113 | Urine pregnancy test kit |  |  |  |
| 114 | Dipsticks for urinalysis |  |  |  |
| 115 | Blood glucose testing using glucometer |  |  |  |
| 116 | Malaria smear test |  |  |  |
| 117 | General microscopy/wet mounts |  |  |  |
| 118 | Haemoglobin testing |  |  |  |
| 119 | Rapid syphilis testing |  |  |  |
| 120 | Blood group and genotype |  |  |  |

**SUPPLY CHAIN**

| **No.** | | **Question** | |  | | | | | |
| --- | --- | --- | --- | --- | --- | --- | --- | --- | --- |
| 121 | Who is the principal person responsible for managing the ordering of medical supplies at this facility? | | | 1. Nurse ___________________________________________ 2. Pharmacy technician _______________________________ 3. Other (specify) ___________________________________ | | | | | |
|  | Which of the following mechanisms is used to determine this facility’s resupply quantities? | | | | 1. **Yes** | | 1. No | | 1. **Don’t know** |
| 122 | The facility itself (pull distribution system) | | | |  | |  | |  |
| 123 | A higher level facility (push distribution system) | | | |  | |  | |  |
| 124 | Other ____________________ (SPECIFY) | | | |  | |  | |  |
| 125 | How are the facility’s resupply quantities determined? | | 1. Formula (any calculation) _______________________ 2. Don’t know ___________________________________ 3. Other means___________________________________ | | | | | | |
| 126 | What is the main source of your routine pharmaceutical commodity supplies? By this I mean who is the direct supplier to your facility? | | 1. National medical stores __________________________ 2. Joint medical stores _____________________________ 3. NGO/donors __________________________________ | | | | | | |
| 127 | How are your pharmaceutical commodity supplies from the main supplier of your routine pharmaceuticals delivered to this facility? | | 1. Supplier delivers to facility _______________________ 2. Facility must arrange delivery to facility ____________ 3. Other (specify)_________________________________ | | | | | | |
|  | Who is responsible for transporting products from central medical stores to your facility? | | | | | 1. **YES** | | 1. **NO** | |
|  |  |  |  |  |  |  | |  | |
| 128 | Local supplier delivers | | | | |  | |  | |
| 129 | Higher level delivers | | | | |  | |  | |
| 130 | This facility collects | | | | |  | |  | |
| 131 | Other ________________________ (SPECIFY) | | | | |  | |  | |
| 132 | For the most recent order, how long did it take between ordering and receiving products? | | 1. 2 weeks to 1 month ___________________________ 2. Between 1 and 2 months _______________________ 3. More than 2 months ___________________________ | | | | | | |

SUPERVISION

| 133 | When was the last time this facility received a supervision visit from the higher level? | 1. This month ____________________ 2. In the last three months___________ 3. More than 3 months ago __________ | |
| --- | --- | --- | --- |
|  | During the supervision visit, did the supervisor assess the following? | 1. **YES** | **2) NO** |
| 134 | Pharmacy (e.g. drug stock out, expiry, records, etc.) |  |  |
| 135 | Staffing (e.g. staff available and training) |  |  |
| 136 | Data (e.g. completeness, quality and timely reporting) |  |  |

**We have now completed all of the questions in this module of the survey. Thank you for your participation.**

| **SECTION 3 CONSTRAINTS AND ENABLERS**  Please write out enabling and constraining issues in delivering the following services and areas   1. Continuing medical education: ____________________________________________   _____________________________________________________________________________  _____________________________________________________________________________  _____________________________________________________________________________  _____________________________________________________________________________   1. Essential medications   _____________________________________________________________________________  _____________________________________________________________________________  _____________________________________________________________________________  _____________________________________________________________________________   1. Funding   _____________________________________________________________________________  ______________________________________________________________________________  ______________________________________________________________________________   1. Maintenance of equipment   ______________________________________________________________________________  ______________________________________________________________________________  _____________________________________________________________________________  _____________________________________________________________________________   1. Others (specify)   __________________________________________________________________________________________________________________________________________________________________________________________________________________________________________________________________________________________________________________________________________________________________________________________________________________________________________________________________________________________________________________________________________________________________________________________________________________________  **We have now completed all of the questions in this module of the survey. Thank you for your participation.** |
| --- |
