# Supplementary material for: How ready is the system to deliver primary healthcare? Results of a primary health facility assessment in Enugu State, Nigeria
Source: Health Policy Plan. 2020 Nov 9;35(Suppl 1):i97–i106. doi: 10.1093/heapol/czaa108 (PMC7649669; doi:10.1093/heapol/czaa108)
Supplement: czaa108_Supplementray_Data [file czaa108_supplementray_data.zip › czaa108-suppl_data/Appendix 2 -Observational checklist.docx]

**OBSERVATIONAL CHECKLIST**

| **No.** | **INFRASTRUCTURE** | **AVAILABLE** | | | **FUNCTIONING** | | |
| --- | --- | --- | --- | --- | --- | --- | --- |
|  |  | 1)Observed | 2)Reported not seen | 3)Not available | 4)Yes | 5)No | 6)Don’t know |
|  | COMMUNICATIONS |  |  |  |  |  |  |
| 1 | Cell phone |  |  |  |  |  |  |
| 2 | Computer |  |  |  |  |  |  |
| 3 | Email or internet access |  |  |  |  |  |  |
|  | TRANSPORT |  |  |  |  |  |  |
| 4 | Ambulance Vehicle |  |  |  |  |  |  |
| 5 | Bicycle |  |  |  |  |  |  |
| 6 | Motorcycle |  |  |  |  |  |  |
| 7 | Fuel for transport |  |  |  |  |  |  |
|  | POWER SUPPLY |  |  |  |  |  |  |
| 8 | Generator |  |  |  |  |  |  |
| 9 | Solar |  |  |  |  |  |  |
| 10 | Grid |  |  |  |  |  |  |
| 11 | Other |  |  |  |  |  |  |
|  | AMENITIES |  |  |  |  |  |  |
| 12 | Piped water |  |  |  |  |  |  |
| 13 | Water from open well |  |  |  |  |  |  |
| 14 | Covered well or borehole |  |  |  |  |  |  |
| 15 | Surface water |  |  |  |  |  |  |
| 16 | Rain water |  |  |  |  |  |  |
| 17 | Tanker truck |  |  |  |  |  |  |
| 18 | Flush toilet |  |  |  |  |  |  |
| 19 | VIP latrine |  |  |  |  |  |  |
| 20 | Pit latrine with slab |  |  |  |  |  |  |
| 21 | Open pit |  |  |  |  |  |  |
| 22 | Composting toilet |  |  |  |  |  |  |
| 23 | Bucket |  |  |  |  |  |  |
| 24 | No toilet facilities/bush/fields |  |  |  |  |  |  |
|  | SHARPS |  |  |  |  |  |  |
| 25 | Burn incinerator |  |  |  |  |  |  |
| 26 | Open burning, no protection |  |  |  |  |  |  |
| 27 | Open burning, protected ground |  |  |  |  |  |  |
| 28 | Dump without burning, flat ground and pit , without protection |  |  |  |  |  |  |
| 29 | Dump without burning, flat ground or protected pit |  |  |  |  |  |  |
| 30 | Remove offsite in covered container |  |  |  |  |  |  |
| 31 | Remove offsite unprotected |  |  |  |  |  |  |
| 32 | Never has sharp waste |  |  |  |  |  |  |
|  | WASTE |  |  |  |  |  |  |
| 33 | Burn Incinerator |  |  |  |  |  |  |
| 34 | Open burning, no protection |  |  |  |  |  |  |
| 35 | Open burning, pit or protected |  |  |  |  |  |  |
| 36 | Dump without burning, flat ground and pit with protection |  |  |  |  |  |  |
| 37 | Dump without burning, flat ground and pit without protection |  |  |  |  |  |  |
| 38 | Remove offsite in covered container |  |  |  |  |  |  |
| 39 | Remove offsite unprotected |  |  |  |  |  |  |

| **No** | **BASIC EQUIPMENT** | **AVAILABLE** | | | **FUNCTIONING** | | |
| --- | --- | --- | --- | --- | --- | --- | --- |
|  |  | 1)Observed | 2)Reported not seen | 3)Not available | 4)Yes | 5)No | 6)Don’t know |
| 40 | Adult weighing scale |  |  |  |  |  |  |
| 41 | Child weighing scale |  |  |  |  |  |  |
| 42 | Infant weighing scale |  |  |  |  |  |  |
| 43 | Measuring tape-height board/stadiometre |  |  |  |  |  |  |
| 44 | Thermometer |  |  |  |  |  |  |
| 45 | Stethoscope |  |  |  |  |  |  |
| 46 | Blood pressure apparatus |  |  |  |  |  |  |
| 47 | Light source |  |  |  |  |  |  |
| 48 | Refrigerator |  |  |  |  |  |  |
| 49 | Latex gloves |  |  |  |  |  |  |
| 50 | Slides |  |  |  |  |  |  |
| 51 | Microscope |  |  |  |  |  |  |
| 52 | Stainless Instrument tray |  |  |  |  |  |  |
| 53 | Hospital bed and mattress |  |  |  |  |  |  |
| 54 | Mackintosh sheet |  |  |  |  |  |  |
| 55 | Dressing trolley |  |  |  |  |  |  |
| 56 | Kidney dish |  |  |  |  |  |  |
| 57 | Tongue depressor |  |  |  |  |  |  |
| 58 | Wall clock |  |  |  |  |  |  |
| 59 | Door name plates |  |  |  |  |  |  |

| No. | Infection control precautions | 1)Observed | 2)Reported, not seen | 3)Not available |
| --- | --- | --- | --- | --- |
| 60 | Clean running water (piped , bucket with tap, or pour pitcher) |  |  |  |
| 61 | Hand washing soap/liquid soap |  |  |  |
| 62 | Alcohol based hand rub |  |  |  |
| 63 | Disposable latex gloves |  |  |  |
| 64 | Waste receptacle (pedal bin) with lid and plastic bin liner |  |  |  |
| 65 | Sharps container |  |  |  |
| 66 | Environmental disinfectant (e.g. chlorine) |  |  |  |
| 67 | Disposable syringes with disposable needles |  |  |  |
| 68 | Auto-disable syringes |  |  |  |

| **No.** | **Infant and child welfare** | **1)Observed** | **2)Reported, not seen** | **3)Not available** |
| --- | --- | --- | --- | --- |
| 69 | Basket with lid for ORS |  |  |  |
| 70 | Spoon measure |  |  |  |
| 71 | Rectal thermometer |  |  |  |
| 72 | Length measure for babies |  |  |  |
| 73 | Vaccine cold box |  |  |  |
| 74 | BCG |  |  |  |
| 75 | OPV |  |  |  |
| 76 | Pentavalent vaccine(valid) |  |  |  |
| 77 | Rotavirus vaccine |  |  |  |
| 78 | Pneumococcal vaccine |  |  |  |
| 79 | Measles vaccine and diluent |  |  |  |
| 80 | Yellow fever |  |  |  |
| 81 | Inactivated polio vaccine |  |  |  |

| **No.** | **Adult wards** | **1)Observed** | **2)Reported, not seen** | **3)Not available** |
| --- | --- | --- | --- | --- |
| 82 | Angle poised lamp |  |  |  |
| 83 | Artery forceps (medium) |  |  |  |
| 84 | Bed pan(stainless steel) |  |  |  |
| 85 | Dissecting forceps |  |  |  |
| 86 | Dressing scissors |  |  |  |
| 87 | Kidney dishes (large) |  |  |  |
| 88 | Galipot (medium) |  |  |  |
| 89 | Forceps jar |  |  |  |
| 90 | Stitch removal/suture scissors |  |  |  |
| 91 | Vaginal speculum, Sims, set of 3 |  |  |  |
|  | LABOUR ROOM |  |  |  |
| 92 | Delivery couch |  |  |  |
| 93 | Dissecting forceps |  |  |  |
| 94 | Enema can |  |  |  |
| 95 | Episiotomy scissors |  |  |  |
| 96 | Foetal stethoscope(Aluminium) |  |  |  |
| 97 | Needle holder |  |  |  |
| 98 | Nail scrubbing brush |  |  |  |
| 99 | Scalpel blade, pack of 100, 4 sizes |  |  |  |
| 100 | Sponge holding forceps |  |  |  |
| 101 | Oral thermometer |  |  |  |
| 102 | Nursery cots |  |  |  |
| 103 | Vacuum extractor, manual |  |  |  |
| 104 | Suction pump |  |  |  |
| 105 | Urinary catheter |  |  |  |
| 106 | Umbilical cord clamp, pack of 100 |  |  |  |
| 107 | Drip stand |  |  |  |
| 108 | Suture kit |  |  |  |
| 109 | Oro-pharyngeal airway |  |  |  |
| 110 | Plastic apron |  |  |  |
|  | LABORATORY |  |  |  |
| 111 | Box, microscope slide, (x100) |  |  |  |
| 112 | Centrifuge, manual |  |  |  |
| 113 | Clam, test tube |  |  |  |
| 114 | Container, sputum, capped |  |  |  |
| 115 | Microscope, binocular |  |  |  |
| 116 | Refrigerator |  |  |  |
| 117 | Scalpel handle |  |  |  |
| 118 | Slides rack |  |  |  |
| 119 | Spirit lamp |  |  |  |
| 120 | Stop watch |  |  |  |
| 121 | Test tube rack |  |  |  |
| 122 | Tray test tube |  |  |  |
| 123 | Bunsen burner |  |  |  |
| 124 | Tripod stand |  |  |  |
| 125 | Wire gauze |  |  |  |
| 126 | Laboratory glass ware |  |  |  |
| 127 | Blood lancets, pack of 200 |  |  |  |
| 128 | Tourniquet |  |  |  |
| 129 | Urine dipstick |  |  |  |
| 130 | Stool specimen bottles |  |  |  |
| 131 | Urine specimen bottles |  |  |  |
| 132 | Haemoglobinometer (sliding type) |  |  |  |
|  | STERILIZATION |  |  |  |
| 133 | Bucket autoclave |  |  |  |
| 134 | Tape dispenser |  |  |  |
| 135 | Scrub brush dispenser |  |  |  |
| 136 | Autoclave tape |  |  |  |
| 137 | Storage cabinet |  |  |  |
| 138 | Sterilizing drums, set of 3 |  |  |  |
|  | CONSULTING CUBICLE |  |  |  |
| 139 | Examination couch |  |  |  |
| 140 | Hammer, reflex |  |  |  |
| 141 | Height measuring stick |  |  |  |
| 142 | Pen torch |  |  |  |
| 143 | Mercurial sphygmomanometer |  |  |  |
| 144 | Stethoscope |  |  |  |
| 145 | Snellen’s chart |  |  |  |
|  | FAMILY PLANNING |  |  |  |
| 146 | Gynae couch |  |  |  |
| 147 | Auvards speculum |  |  |  |
| 148 | Tenaculum |  |  |  |

**THE PRIMARY HEALTH CARE ESSENTIAL DRUG LIST FOR PRIMARY HEALTH CENTRES**

**Group (I) Formulation**

| No. | **ESSENTIAL DRUG LIST** | **OBSERVED AVAILABLE** | | | **NOT OBSERVED** | | |
| --- | --- | --- | --- | --- | --- | --- | --- |
|  |  | 1) At least one valid | 2)Available not valid | | 3)Reported available but not seen | 4)Not available today | 5)Never available |
| 149 | Lidocaine - Topical,injection |  |  | |  |  |  |
|  | ANALGESICS |  |  | |  |  |  |
| 150 | Acetylsalicylic Acid* - Tablet*Not for children |  |  | |  |  |  |
| 151 | Paracetamol - Oral liquid, tablet |  |  | |  |  |  |
|  | ANTI-ALLERGICS |  |  | |  |  |  |
| 152 | Chlorphenamine - Oral liquid, tablet |  |  | |  |  |  |
| 153 | Epinephrine (Adrenaline) – Injection |  |  | |  |  |  |
| 154 | Promethazine - Tablet, oral liquid |  |  | |  |  |  |
|  | ANTICONVULSANTS |  |  | |  |  |  |
| 155 | Diazepam – Injection |  |  | |  |  |  |
| 156 | Paraldehyde** - Injection |  |  | |  |  |  |
| 157 | Phenobarbital – Tablet |  |  | |  |  |  |
|  | ANTIDOTES |  |  | |  |  |  |
| 158 | Atropine – Injection |  |  | |  |  |  |
| 159 | Charcoal (activated) – Powder |  |  | |  |  |  |
|  | ANTI-INFECTIVE DRUGS |  |  | |  |  |  |
| 160 | Antibacterial drugs |  |  | |  |  |  |
| 161 | Amoxicillin – Capsule |  |  | |  |  |  |
| 162 | Benzathine Penicillin – Injection |  |  | |  |  |  |
| 163 | Benzylpenicillin – Injection |  |  | |  |  |  |
| 164 | Co-trimoxazole - Tablet, oral liquid |  |  | |  |  |  |
| 165 | Erythromycin – Tablet |  |  | |  |  |  |
| 166 | Gentamicin – Injection |  |  | |  |  |  |
| 167 | Nitrofurantoin – Tablet |  |  | |  |  |  |
| 168 | Phenoxymethylpenicillin – Tablet |  |  | |  |  |  |
| 169 | Streptomycin – Injection |  |  | |  |  |  |
| 170 | Tetracycline* - Capsule*Not recommended for children and pregnant women |  |  | |  |  |  |
|  | ANTILEPROSY DRUGS |  |  | |  |  |  |
| 171 | Clofazimine – Capsule |  |  | |  |  |  |
| 172 | Dapsone – Tablet |  |  | |  |  |  |
| 173 | Rifampicin - Capsule or tablet |  |  | |  |  |  |
|  | AMOEBICIDE |  |  | |  |  |  |
| 174 | Metronidazole – Tablet |  |  | |  |  |  |
|  | ANTIHELMINTICS |  |  | |  |  |  |
| 175 | Mebendazole – Tablet |  |  | |  |  |  |
| 176 | Praziquantel – Table |  |  | |  |  |  |
| 177 | Pyrantel - Oral liquid, tablet |  |  | |  |  |  |
|  | ** Marked for deletion |  |  | |  |  |  |
|  | ANTIFILARIAL |  |  | |  |  |  |
| 178 | Diethylcarbamazine – Tablet |  |  | |  |  |  |
|  | ANTIMALARIALS |  |  | |  |  |  |
| 179 | Artemether + lumefantrine - Oral liquid, tablet |  |  | |  |  |  |
| 180 | Artesunate – Suppositories |  |  | |  |  |  |
| 181 | Artesunate + amodiaquine – Tablet |  |  | |  |  |  |
| 182 | Quinine - Injection*  *Intramuscular, for pre-referral treatment only |  | |  |  |  |  |
| 183 | Pyrimethamine + sulfadoxine - Tablet, oral liquid |  |  | |  |  |  |

**THE PRIMARY HEALTH CARE ESSENTIAL DRUG LIST**

**FOR PRIMARY HEALTH CENTRES**

**(II)Group Formulation**

| No. | **ESSENTIAL DRUG LIST** | **OBSERVED AVAILABLE** | | **NOT OBSERVED** | | |
| --- | --- | --- | --- | --- | --- | --- |
|  |  | 1) At least one valid | 2)Available not valid | 3)Reported available but not seen | 4)Not available today | 5)Never available |
|  | ANTI-TUBERCULOSIS DRUGS |  |  |  |  |  |
| 184 | Ethambutol – Tablet |  |  |  |  |  |
| 185 | Isoniazid – Tablet |  |  |  |  |  |
| 186 | Pyrazinamide – Tablet |  |  |  |  |  |
| 187 | Rifampicin - Capsule, tablet |  |  |  |  |  |
|  | **ANTISEPTICS AND DISINFECTANTS** |  |  |  |  |  |
| 188 | Benzoin – Compound tincture |  |  |  |  |  |
| 189 | Chlorhexidine – Solution |  |  |  |  |  |
| 190 | Iodine – Solution |  |  |  |  |  |
| 191 | Methylated spirit – Solution |  |  |  |  |  |
| 192 | Sodium hypochlorite – Solution |  |  |  |  |  |
|  | **DERMATOLOGICAL DRUGS** |  |  |  |  |  |
| 193 | Benzoic acid+salicylic acid |  |  |  |  |  |
| 194 | (Whitfield's) – Ointment |  |  |  |  |  |
| 195 | Benzoyl peroxide - Cream or gel |  |  |  |  |  |
| 196 | Benzyl benzoate – Emulsion |  |  |  |  |  |
| 197 | Calamine – Lotion |  |  |  |  |  |
| 198 | Gentamicin – Ointment |  |  |  |  |  |
| 199 | Methyl salicylate – Ointment |  |  |  |  |  |
| 200 | Neomycin+Bacitracin - Ointment, powder |  |  |  |  |  |
| 201 | Nystatin - Ointment, cream |  |  |  |  |  |
| 202 | Zinc oxide – Ointment |  |  |  |  |  |
|  | **DRUGS AFFECTING THE BLOOD** |  |  |  |  |  |
| 203 | Ferrous salts - Oral liquid, tablet |  |  |  |  |  |
| 204 | Folic acid – Tablet |  |  |  |  |  |
|  | **DIAGNOSTIC AGENT** |  |  |  |  |  |
| 205 | Tuberculin - Injection, PPD |  |  |  |  |  |
|  | **DRESSINGS AND MEDICAL DEVICES** |  |  |  |  |  |
| 206 | Absorbent gauze bandages |  |  |  |  |  |
| 207 | Cotton wool (absorbent) |  |  |  |  |  |
| 208 | Disposable gloves, |  |  |  |  |  |
| 209 | Disposable syringes - 5 mL with needles (19, 21 Gauge) |  |  |  |  |  |
| 211 | Disposable syringes - 2 mL with needles (19, 21 Gauge) |  |  |  |  |  |
|  | **EAR, NOSE AND THROAT DRUGS** |  |  |  |  |  |
| 212 | Chloramphenicol - Ear drops |  |  |  |  |  |
|  | **GASTRO-INTESTINAL DRUGS** |  |  |  |  |  |
| 213 | Hydrocortisone + lidocaine – Suppository |  |  |  |  |  |
| 214 | Hyoscine N-butylbromide – Tablet |  |  |  |  |  |
| 215 | Magnesium Sulphate – Injection |  |  |  |  |  |
| 216 | Magnesium trisilicate - Compound tablet,  oral liquid |  |  |  |  |  |
| 217 | Misoprostol – Tablets |  |  |  |  |  |
| 218 | Oral Rehydration Salts |  |  |  |  |  |
| 219 | Senna – Tablet |  |  |  |  |  |
| 220 | Zinc - Oral liquid, tablet |  |  |  |  |  |
|  | **HORMONES AND SYNTHETIC SUBSTITUTES** |  |  |  |  |  |
| 221 | Barrier methods - Condoms with or without spermicide |  |  |  |  |  |
| 222 | Oral contraceptives – Tablet |  |  |  |  |  |

**THE PRIMARY HEALTH CARE ESSENTIAL DRUG LIST**

**FOR PRIMARY HEALTH CENTRES**

**(III)**

| No. | **ESSENTIAL DRUG LIST** | **OBSERVED AVAILABLE** | | **NOT OBSERVED** | | |
| --- | --- | --- | --- | --- | --- | --- |
|  |  | 1) At least one valid | 2)Available not valid | 3)Reported available but not seen | 4)Not available today | 5)Never available |
| 223 | Poliomyelitis vaccine - Oral liquid |  |  |  |  |  |
| 224 | Rabies immunoglobulin – Injection |  |  |  |  |  |
| 225 | Tetanus vaccine – Injection |  |  |  |  |  |
|  | **OPHTHALMOLOGICAL DRUGS** |  |  |  |  |  |
| 226 | Chloramphenicol - Eye drops,  Ointment |  |  |  |  |  |
| 227 | Chlortetracycline - Eye ointment |  |  |  |  |  |
|  | **OXYTOCIC** |  |  |  |  |  |
| 228 | Oxytocine - |  |  |  |  |  |
| 229 | Ergometrine - Tablet, injection |  |  |  |  |  |
|  | **PSYCHOTHERAPEUTIC DRUG** |  |  |  |  |  |
| 230 | Chlorpromazine – Injection |  |  |  |  |  |
|  | **RESPIRATORY DRUGS** |  |  |  |  |  |
| 231 | Beclomethasone – Inhaler |  |  |  |  |  |
| 232 | Salbutamol - Tablet, inhaler |  |  |  |  |  |
|  | **VITAMINS AND MINERALS** |  |  |  |  |  |
| 233 | Ascorbic Acid (vitamin C) – Tablet |  |  |  |  |  |
| 234 | Calcium gluconate – Injection |  |  |  |  |  |
| 235 | Calcium salts – Tablet |  |  |  |  |  |
| 236 | Folic acid – Tablet |  |  |  |  |  |
| 237 | Vitamin A – Capsule |  |  |  |  |  |
|  | **MISCELLANEOUS** |  |  |  |  |  |
| 238 | Water for injection – Injection |  |  |  |  |  |
| 239 | Spatulas |  |  |  |  |  |

**DRUG LIST FOR NON COMMUNICABLE DISEASES**

|  |  | 1) At least one valid | 2)Available not valid | 3)Reported available but not seen | 4)Not available today | 5)Never available |
| --- | --- | --- | --- | --- | --- | --- |
| 240 | Metformin cab/tab |  |  |  |  |  |
| 241 | Insulin regular injection |  |  |  |  |  |
| 242 | Glucose 50% injection |  |  |  |  |  |
| 243 | ACE inhibitor (e.g. enalapril, lisinopril, ramipril, perindopril) |  |  |  |  |  |
| 244 | Thiazide (e.g. hydrochlorothiazide) |  |  |  |  |  |
| 245 | Beta blocker (e.g.bisoprolol, metoprolol, carvedilol, atenolol) |  |  |  |  |  |
| 246 | Calcium channel blocker (e.g. amlodipine) |  |  |  |  |  |
|  | Aspirin cap/tab |  |  |  |  |  |
| 247 | Beclomethasone inhaler |  |  |  |  |  |
| 248 | Prednisolone cap/tab |  |  |  |  |  |
| 249 | Hydrocortisone injection |  |  |  |  |  |
| 250 | Epinephrine injection |  |  |  |  |  |
| 251 | Furosemide cap/tab |  |  |  |  |  |
| 252 | Glibenclamide cap/tab |  |  |  |  |  |
| 253 | Gliclazide tablet or glipizide tablet |  |  |  |  |  |
| 254 | Glyceryl trinitrate sublingual tablet |  |  |  |  |  |
| 255 | Ibuprofen tablet |  |  |  |  |  |
| 256 | Isosorbide dinitrate tablet |  |  |  |  |  |
| 257 | Omeprazole tablet or alternative such as pantoprazole, rabeprazole |  |  |  |  |  |
| 258 | Paracetamol cap/tab (adult oral formulation) |  |  |  |  |  |
| 259 | Salbutamol inhaler |  |  |  |  |  |
| 260 | Simvastatin tablet or other statin e.g. atorvastatin, pravastatin, fluvastatin |  |  |  |  |  |
| 261 | Spironolactone tablets |  |  |  |  |  |
